# A hypercubic Mk model framework for capturing reversibility in disease, cancer, and evolutionary accumulation modelling

**DOI:** 10.1093/bioinformatics/btae737

**Published:** 2024-12-12

**Authors:** Iain G Johnston, Ramon Diaz-Uriarte

**Affiliations:** Department of Mathematics, University of Bergen, Realfagbygget, Bergen 5007, Norway; Computational Biology Unit, University of Bergen, Thormøhlensgate 55, Bergen 5008, Norway; Department of Biochemistry, School of Medicine, Universidad Autonoma de Madrid, Madrid 28029, Spain; Instituto de Investigaciones Biomedicas Sols-Morreale (IIBM), CSIC-UAM, Madrid 28029, Spain

## Abstract

**Motivation:**

Accumulation models, where a system progressively acquires binary features over time, are common in the study of cancer progression, evolutionary biology, and other fields. Many approaches have been developed to infer the accumulation pathways by which features (e.g. mutations) are acquired over time. However, most of these approaches do not support reversibility: the loss of a feature once it has been acquired (e.g. the clearing of a mutation from a tumor or population).

**Results:**

Here, we demonstrate how the well-established Mk model from evolutionary biology, embedded on a hypercubic transition graph, can be used to infer the dynamics of accumulation processes, including the possibility of reversible transitions, from data which may be uncertain and cross-sectional, longitudinal, or phylogenetically/phylogenomically embedded. Positive and negative interactions between arbitrary sets of features (not limited to pairwise interactions) are supported. We demonstrate this approach with synthetic datasets and real data on bacterial drug resistance and cancer progression. While this implementation is limited in the number of features that can be considered, we discuss how this limitation may be relaxed to deal with larger systems.

**Availability and implementation:**

The code implementing this setup in R is freely available at https://github.com/StochasticBiology/hypermk.

## Graphical Abstract

**Graphical Abstract:**
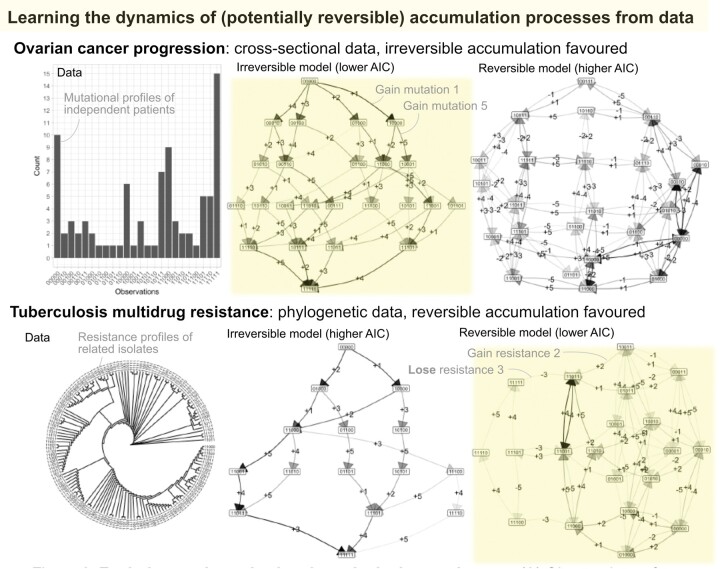

## 1 Introduction

Many systems of interest in biology, medicine, and beyond involve the progressive, random, coupled accumulation of different binary features over time. Such features—variously called traits or characters—may be, e.g. the presence or absence of particular mutations in a tumor (Beerenwinkel *et al.* 2015, Schwartz and Schäffer 2017, Schill *et al.* 2020, Diaz-Uriarte and Johnston 2024), susceptibility or resistance to particular drugs in evolving pathogens (Beerenwinkel *et al.* 2005, Greenbury *et al.* 2020, Moen and Johnston 2023, Renz *et al.* 2024), the presence or absence of genes in evolving genomes (Johnston and Williams 2016), or the presentation or absence of particular clinical symptoms in patients (Johnston *et al.* 2019, Schill *et al.* 2024). In all these cases, the study of the dynamics by which features are acquired—the trajectories or pathways of accumulation—are of interest in both describing the scientific system and predicting its future behavior.

The field of accumulation modelling considers how sets of discrete, usually binary, features are acquired over time in a system. Many approaches have been developed in the cancer literature, where features are often driver mutations, and samples are independent patients (Beerenwinkel *et al.* 2015, Montazeri *et al.* 2016, Schill *et al.* 2020, Angaroni *et al.* 2022, Diaz-Uriarte and Herrera-Nieto 2022, Diaz-Uriarte and Johnston 2024, Schill *et al.* 2024) or, recently, trees reflecting the development of tumors over time (Caravagna *et al.* 2018, Luo *et al.* 2023, Aga *et al.* 2024). There are 2^*L*^ possible patterns of the presence or absence for each of *L* features, and transitions are usually considered to correspond to the acquisition of exactly one feature. Here, a system may be in one of 2^*L*^ states, and transitions between any pair *i* and *j* of these states occur with a rate *r_ij_*, which can be nonzero only if *j* differs from *i* by the presence of one feature.

**Figure 1.**
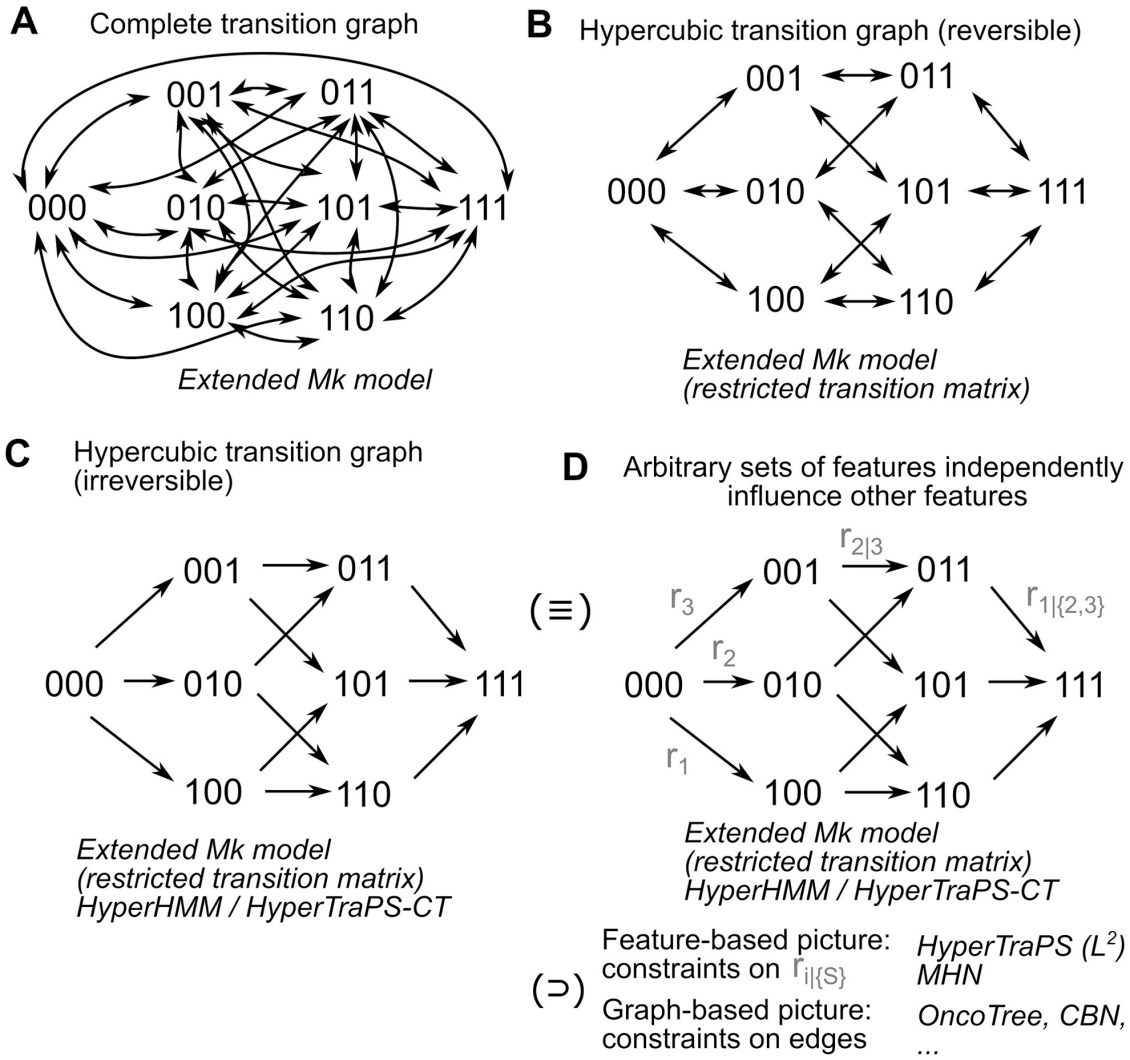
State spaces and features in (reversible) accumulation and Mk models. The state space and supported transitions in several accumulation model pictures for an *L* = 3 feature system. Italics give the names of some approaches using each picture. (A) Unrestricted dynamics, where every state can transition to every other. (B) Reversible stepwise dynamics. (C) Irreversible stepwise dynamics. (D) Different accumulation modelling approaches are equivalent to the irreversible stepwise dynamics picture, and may assume independent rates, impose structure in the transition rates (modelling the influence of each feature on each other), or support only a subset of transitions (modelling dependencies in feature acquisitions). More detail in Supplementary Fig. 1.

The Markov k-state (Mk) model (Pagel 1994, Lewis 2001) is widely used in evolutionary biology to infer the dynamics by which features (usually called characters, in this context) change between discrete states on a phylogeny. Here, every instance of a system can occupy one of *k* discrete states, with Markovian transitions allowed between these states (Fig. 1A). As an evolutionary process branches into different lineages, descendants inherit their ancestor’s state and may transition to other states independently of their sister lineages. Different versions of the model place, or relax, different constraints on the rates of the transitions between states, which are parameters of the model. The original real-world example is how two binary traits, multiple mating partners and posterior decoration, co-evolve in primates (Pagel 1994). More generally in the Mk model (Boyko and Beaulieu 2021), a system may be in one of *k* states, and transitions between any pair *i* and *j* of these states occur with a rate *r_ij_* for a given instance of the system.

As discussed previously (Moen and Johnston 2023), a mathematical connection exists between these two approaches, clearly visible from the final sentences of the preceding two paragraphs. If *k *=* *2^*L*^ and the transition rates for the Mk model are constrained to be zero for the necessary pairs of states (those that differ by the acquisition of more than one change), the set of supported transitions in the Mk model are identical with those in the accumulation model (Fig. 1C and D).

Consider now a *reversible* accumulation model, which is the same as the accumulation model, but where *r_ij_* is now also allowed to be nonzero if *j* differs from *i* by the *absence* of one feature. In other words, the system can transition by acquiring *or losing* individual features. This reversibility challenges existing approaches designed to model accumulation processes, because an infinite set of accumulation pathways now in principle exists between any two states (involving arbitrarily long sequences of feature gain-loss pairs). However, this reversible picture still corresponds to a subset of the more general Mk model (Fig. 1B). Indeed, using a (potentially hidden) Markov modelling framework to explore the coupled evolution of many binary traits is a central idea behind evolutionary analysis packages like *corHMM* (Boyko and Beaulieu 2021). Here, we will show that existing methods from the evolutionary literature for inferring parameters in the Mk model can readily be applied to this reversible accumulation picture, allowing analysis of this problem.

## 2 Materials and methods

### 2.1 State spaces in (reversible) accumulation and Mk models

Consider a state space **S** = {*S*_0_, …, $S_{2^{L}-1}$}, consisting of 2^*L*^ states. For convenience, we set the index of a state to the decimal value of a binary string of length *L*, corresponding to the absence (0) or presence (1) of each of the *L* features in that state (e.g. for *L *=* *3, 000 ≡ 0, 001 ≡ 1, 010 ≡ 2, …, 110 ≡ 6, 111 ≡ 7). This is a general procedure for coding combinations of binary states as multistate characters that has been used before with the Mk model (e.g. in Boyko and Beaulieu (2021: 470)). This transformation allows us to seamlessly use the Mk model for accumulation processes that include the cross-sectional case, and that, then, allow us to model both irreversible and reversible cases.

We label the transition rate from the state with index *i* to the state with index *j* as *r_ij_*. A general accumulation-only model allows *r_ij_* > 0 if and only if *j* *−* *i *= 2^*m*^, where *m* is a nonnegative integer. In other words, transitions are only supported from *i* to *j* if *i* lacks exactly one feature that *j* possesses. The corresponding transition graph for this model takes the form of an *L*-dimensional hypercube with single directed edges (Fig. 1C). A reversible accumulation model allows *r_ij_* > 0 if and only if *j* *−* *i *=* *2^*m*^ or *i* *−* *j *=* *2^*m*^. Hence, two (different) nonzero rates can correspond to the transition from *i* to *j* (gaining a feature) and from *j* to *i* (losing that feature). Now the transition graph is an *L*-dimensional hypercube with pairs of opposing directed edges between vertices (Fig. 1B).

Now consider an Mk model with *k *=* *2^*L*^ states. The most general picture (called the extended Mk model) allows *r_ij_* > 0 for all *i* and *j*. Hence, the transition graph is a complete graph, with pairs of opposing directed edges between every pair of vertices (Fig. 1A). Clearly, both the transition graphs for the accumulation-only and reversible accumulation models are subgraphs of the extended Mk model transition graph, and can be captured by constraining the required set of edges in the Mk model to be zero. We can then use these specific parameterizations of the Mk model as an accumulation model, as they allow transitions only between those states that an accumulation model would support.

More precisely, an *extended Mk model* is generally parameterized by a *k* × *k* matrix representing transition rates *r_ij_* from every state *i* to every other state *j*. A *reversible accumulation model* describing *L* features can be represented as a *k* × *k* matrix (where *k *=* *2^*L*^) representing transition rates *r_ij_*, where *r_ij_* > 0 if and only if *j* *−* *i *=* *2^*m*^ or *i* *−* *j *=* *2^*m*^ for nonnegative integer *m*. An *irreversible accumulation model* can similarly be represented as a *k* × *k* matrix (where *k *=* *2^*L*^) representing transition rates *r_ij_*, where *r_ij_* > 0 if and only if *j* *−* *i *=* *2^*m*^ for nonnegative integer *m*. If we write {*θ_eMk_*}, {*θ_rev_*}, {*θ_irrev_*} respectively for the sets of possible parameterizations of these models, it can readily be seen that {*θ_irrev_*} ⊂ {*θ_rev_*} ⊂ {*θ_eMk_*}, simply by progressively relaxing the set of elements constrained to be zero.

### 2.2 Relationships between observations in (reversible) accumulation and Mk models

We now consider the forms of data that can be used in the different modelling cases. Accumulation modelling often considers cross-sectional, independently observed samples (motivated by the cancer literature, where data have traditionally consisted of single observations for independent patients). Some developments including HyperTraPS (Johnston and Williams 2016, Greenbury *et al.* 2020. Johnston and Røyrvik 2020) and recent expansion HyperTraPS-CT (Aga *et al.* 2024), HyperHMM (Moen and Johnston 2023), and TreeMHN (Luo *et al.* 2023) allow observations to be connected *via* a tree. This may exactly be a phylogenetic tree in evolutionary applications, or a phylogenomic tree (corresponding to different cell lineages evolving within a patient or within a tumor) for disease cases. In all cases, the typical assumption is that a precursor or ancestral state exists which has not acquired any of the features considered (the patient before any cancer developed, for example).

The Mk model considers phylogenetically embedded data, where observations are often taken to represent the tips (terminal vertices) on a phylogenetic tree. This situation is motivated by the evolutionary biology literature, where we often observe properties of modern-day samples that are historically connected by their evolutionary relationship. The state of the root of the tree, and of internal nodes, is not in general known precisely (being in the unobserved evolutionary past) and must be inferred if required.

**Figure 2.**
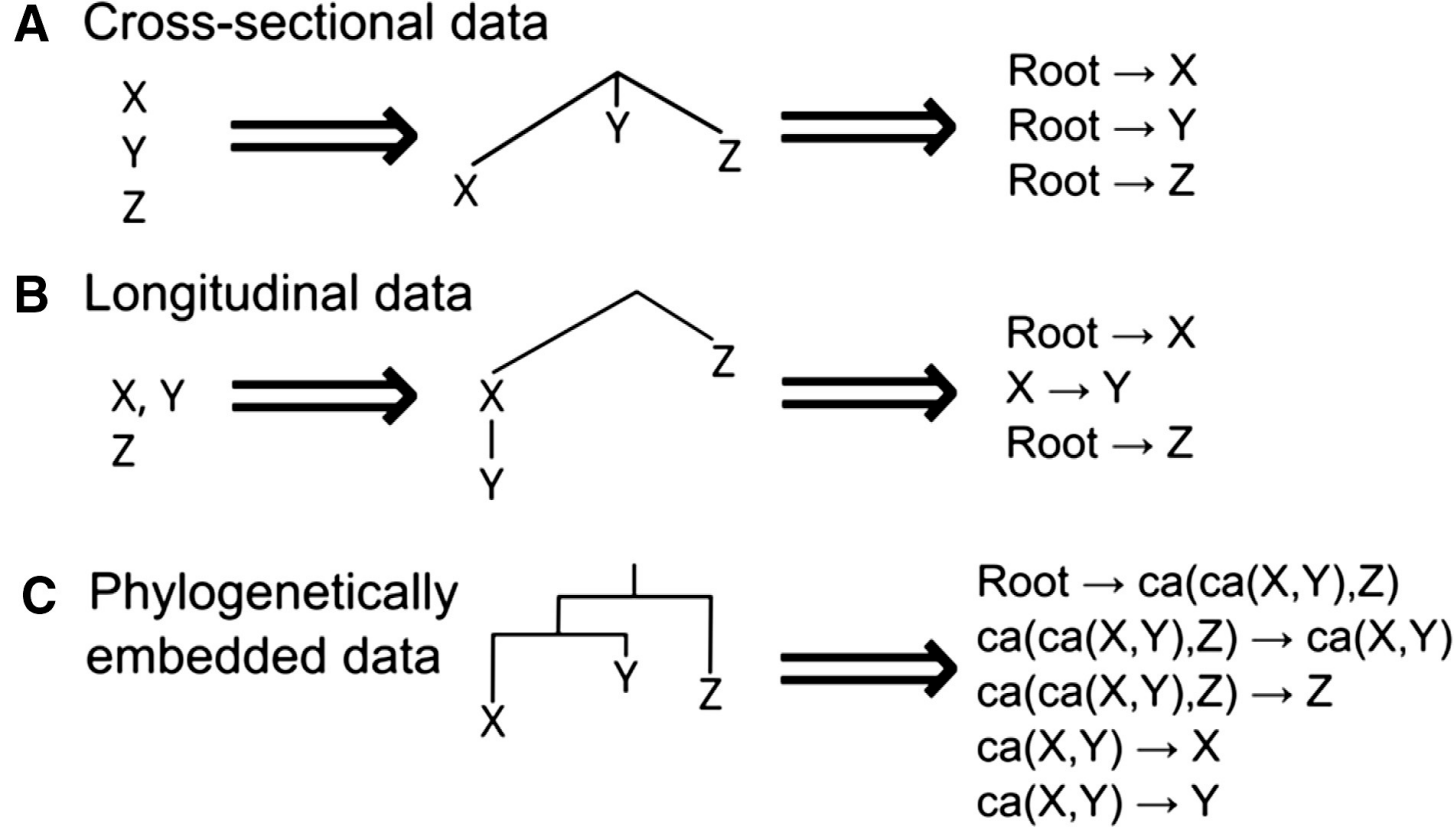
Data types in accumulation modelling, and casting them as trees. (A) Cross-sectional data. Independent observations *X*, *Y*, *Z* can be represented as independent lineages branching from an initial state (the root of a polychotomous tree). The source data are then the set of independent transitions from the root state to each observation. (B) Longitudinal data. Each independent series can be represented as an independent lineage branching from an initial state, with progressive observations in a series following that lineage. The source data are then transited from the root to each lineage’s ancestor, then transited within the lineage. (C) Phylogenetic data. With a phylogeny, transitions are independent ancestor–descendant pairs. Ancestral states (ca, common ancestor) can be reconstructed using phylogenetic methods and/or invoking assumptions about the dynamics of the particular evolutionary process.

These various cases can be collected under an overall umbrella (Fig. 2). A dataset is connected by a set of *n_tree_* trees. In the cross-sectional case, we can either picture *n_tree_* = 1 “star” tree connecting a single root node with state 0 to *n* independent observations, or *n_tree_* = *n* “stump” trees, each connecting a root at state 0 to a single descendant observation; for cross-sectional data, the single star tree, with possibly different branch lengths is appropriate where different branch lengths reflect different times to sample. For the other cases, the trees (phylogenetic or phylogenomic) connecting observations come directly from the data.

We will refer to this picture, using a reversible or irreversible hypercubic transition graph in conjunction with the Mk model, as the “hypercubic Mk model” or “HyperMk” for short, but emphasize that this refers to the model structure and not a particular numerical implementation. As we discuss below, different implementations are possible and further refinements for the HyperMk case specifically are certainly feasible.

### 2.3 Numerical implementation

Given data, inference of the transition parameters in an Mk model can be performed *via* likelihood maximization, often using Felstenstein’s pruning algorithm (Felsenstein 1973), a dynamic programming approach recursing up the levels of the tree and considering conditional dependencies on ancestral states. Many specific software implementations exist for this process (see below and Supplementary Figs S1 and S2). We will focus on the *castor* package in R (Louca and Doebeli 2018, Louca and Pennell 2020) as an easily-implementable, open-source, solution with the flexibility to deal with the different HyperMk structures described above. The Mk fitting routine in *castor* (function fit_mk) supports inference using multiple trees and constraints on transition parameters, as well as the specification of prior states for unobserved vertices.

Data that are already embedded on one or more trees can naturally be passed to *castor*. Cross-sectional data could, in principle, be passed as a set of “stump” trees with one root and one tip. However, we found that this protocol challenged the numerical behavior of the fitting procedure. Instead we adopt the following protocol. For every observation, we construct a tree with one root and two tips. Using the capacity of *castor* to support prior distributions on vertices, we enforce that one tip must have the state corresponding to observation *i*, and allow the second tip to be completely unconstrained (uniform prior over all states). In this way, we produce a branching tree structure that is readily analyzed by *castor* without introducing any artefacts into the dataset.

Several other R packages offer the ability to fit Mk models of a given structure. The *corHMM* package (Boyko and Beaulieu 2021) offers a powerful interface for inferring transition parameters of (hidden) Markov models, which can readily be used to implement this hypercubic Mk model picture. The representation of states as binary strings is an explicit focus of that research, making the hypercubic modelling framework particularly easy to implement. However, we could not find a straightforward way of analyzing cross-sectional or multiple independent trees using this package. *phytools* (Revell 2012) also includes functionality for fitting Mk models and allows the natural inclusion of cross-sectional observations by considering a “star” phylogeny (polychotomous independent branches from an initial state). Here, we are not using *phytools*' fitPagel function to model correlated evolution because that function only allows for two traits, and because our transformation of combinations of binary traits into multistate characters is the standard procedure for analyzing with multiple, possibly correlated characters (see Section 2, Boyko and Beaulieu 2021). However, the inclusion of uncertain data and the adjustment of the parameters of the underlying optimization process were less straightforward in this approach than in *castor*; we also found that some observations were apparently ignored in some cases. We underline that these—and likely more—alternatives may readily be used for many of the case studies in this report; Supplementary Fig. S2 demonstrates their application to a simple case study, and the code repository includes examples of using these different libraries as the core solver.

The code implementing this setup in R (R Core Team 2022) is freely available at https://github.com/StochasticBiology/hypermk and in addition to the libraries described above also uses libraries *ape* (Paradis and Schliep 2019) and *phangorn* (Schliep 2011) for modelling trees, *ggplot2* (Wickham 2016), *ggraph* (Pedersen 2020), *igraph* (Csardi and Nepusz 2006), *ggtree* (Yu *et al.* 2017), and *ggpubr* (Kassambara 2020) for visualization.

### 2.4 Pruning and model comparison

In both the irreversible and reversible models, it can be (and often is) the case that many edges in the hypercubic transition network reflect transitions which do not contribute to the likelihood. For example, in the irreversible case, if 000–001 is the only transition from the initial state 000, no transitions from 100 or 010 will ever contribute to the likelihood, as those source states are unreachable from the initial state. We implemented a “pruning” method to remove parameters associated with these negligible transitions (Supplementary Fig. S3). Pruning—removing particular edges from the hypercubic transition graph—can readily be used to enforce a particular subset of dynamics in a model (Supplementary Fig. S3B), but can also be used to simplify models by removing these extraneous parameters (Supplementary Fig. S3C). From a fitted model, we simulate dynamics on the transition network and track the probability associated with each transition. We then explicitly remove edges with probability below a threshold (by default 0.01, reduced to 10^−5^ for the tuberculosis case study to avoid removing edges that contribute to rare observations), and refit the model without these parameters. For all cases we confirm that the “pruned” likelihood is not detectably different from the original likelihood, confirming that we have not removed edges that contribute to the likelihood. The “pruned” version of that model, with a likelihood and parameter count, can be used in comparisons *via* model selection criteria; we use the Akaike information criterion (AIC) by default (Supplementary Fig. S3).

## 3 Results

### 3.1 Synthetic case studies

To test the capacity of the Mk model picture to capture accumulation models, we construct a set of illustrative case studies with synthetic data. We first simulate a phylogeny using a birth–death process and then simulate an evolutionary process on this phylogeny, involving *L *=* *5 features; the different instances discussed below differ in the accumulation model. In the first, irreversible, instance, these features are acquired in the deterministic order 00000–10000–11000–11100–11110–11111. The corresponding tree is shown in Supplementary Fig. S4A. The Mk model, with parameters restricted to support irreversible accumulation, immediately identifies the generating pathway (Supplementary Fig. S4B and C). For the illustrated dataset, the AIC of the HyperMk model with irreversible dynamics is 125.8, and that with reversible dynamics is 142.7; the lower AIC for the irreversible model reflects the fact that the less complex irreversible model is sufficient to capture these accumulation-only dynamics.

**Figure 3.**
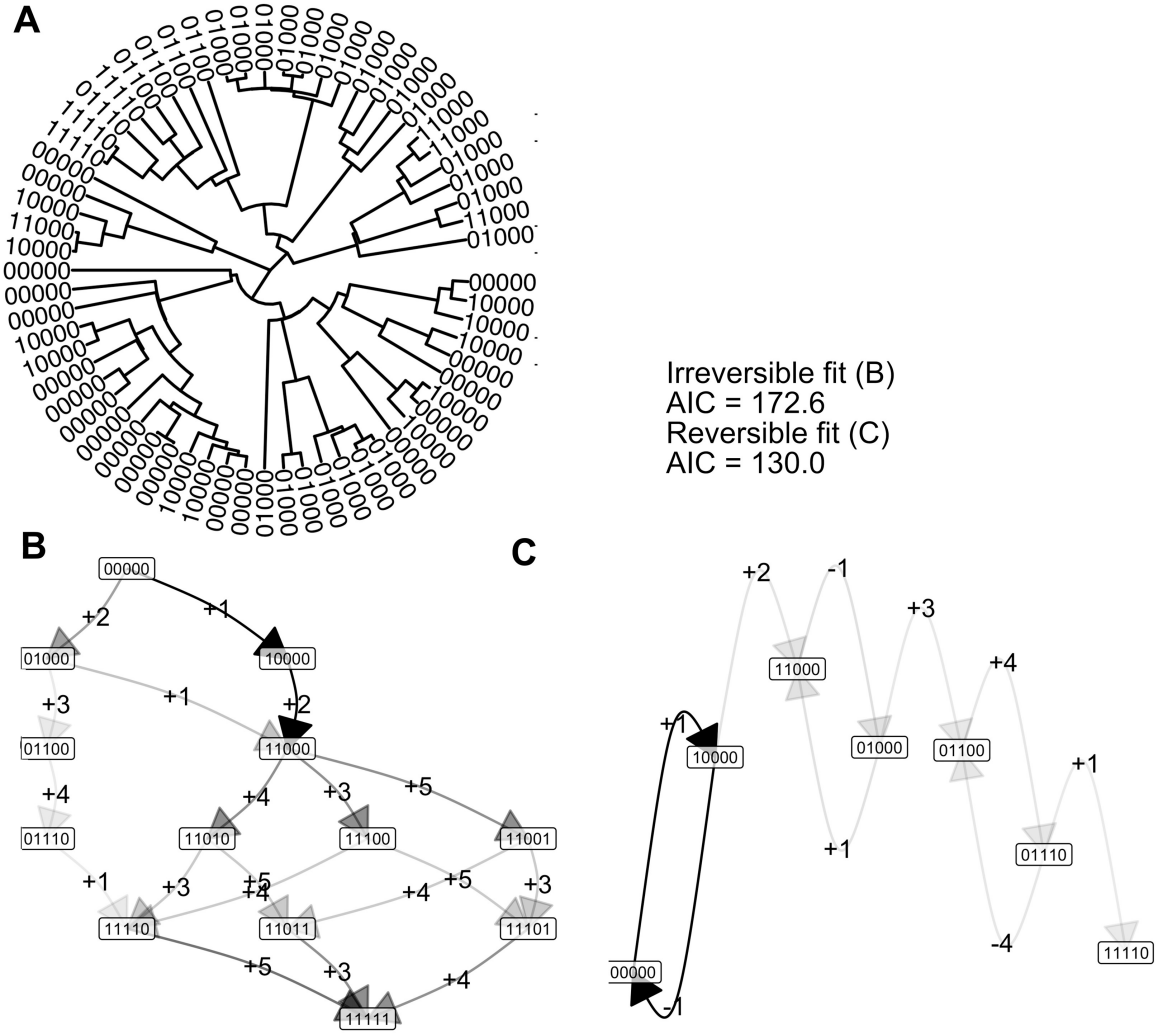
Capturing irreversible and reversible accumulation dynamics with the Mk model. (A) Observations of different states of a reversible process on the tips of a 64-node randomly-simulated tree. (B) Inferred transition graph for these data, using an irreversible model. Node labels give different states; edge labels give the feature that is gained (+) or lost (−) at each transition, but in this irreversible case no feature losses (−) are allowed. The darkness of an edge gives its rate: darker edges have higher associated transition rates. (C) Inferred transition graph for these data using a reversible model. The AIC values for the full fitted model, and for the simplified model discarding all edges that do not support flux from the 00000 state (i.e. edges with two or more transitions), are given: the AIC for the irreversible model is lower, reflecting the irreversible generating process. More detail in Supplementary Fig. S4.

Next, we allow the first feature to undergo reversion, so that transitions involving the loss of the first feature are allowed from any state (Fig. 3A). The corresponding outcomes are shown in Fig. 3B and C (and compared with the irreversible case in Supplementary Fig. S4). Now, the reversible model has a lower AIC (130.0 compared to 172.6), reflecting the fact that a model allowing reversible transitions better capture the observations than the accumulation-only model.

To demonstrate HyperMk’s ability to capture positive and negative influences between traits, and to work with cross-sectional as well as phylogenetically-embedded observations, we next consider an *L *=* *4 case where two pathways are supported: 0000–0001–0011–0111–1111 and 0000–1000–1100–1110–1111. These two pathways are mutually repressing, so that the first step taken from 0000 completely determines the subsequent dynamics. We use the protocol described in Section 2 to represent observations from this system as a collection of trees that can be analyzed by *castor*, and show the results in Supplementary Fig. S5A–C. As expected, given the lack of restrictions placed on the transitions, HyperMk readily captures the cross-repressing nature of these pathways.

The Mk model can naturally account for uncertainty in observations, and uncertainty in the inferred transition parameters can be quantified via resampling, e.g. through using the bootstrap. In the fit routine in the *castor* package, uncertain observations are included by specifying “tip priors”: *P_is_* is the likelihood of the observed state of tip *i* given that it was truly in state *s*. We demonstrate inference using data generated from the single pathway model (Supplementary Fig. S4A–C) with uncertainty in Supplementary Fig. S5D–F. For the irreversible model, HyperMk readily identifies a parsimonious generating mechanism. For the reversible model, the core of this mechanism is identified but with corresponding uncertainty. This uncertainty is manifest in the nonzero probabilities of transitions involving states that are not generated by this process.^1^

### 3.2 Cancer progression

**Figure 4.**
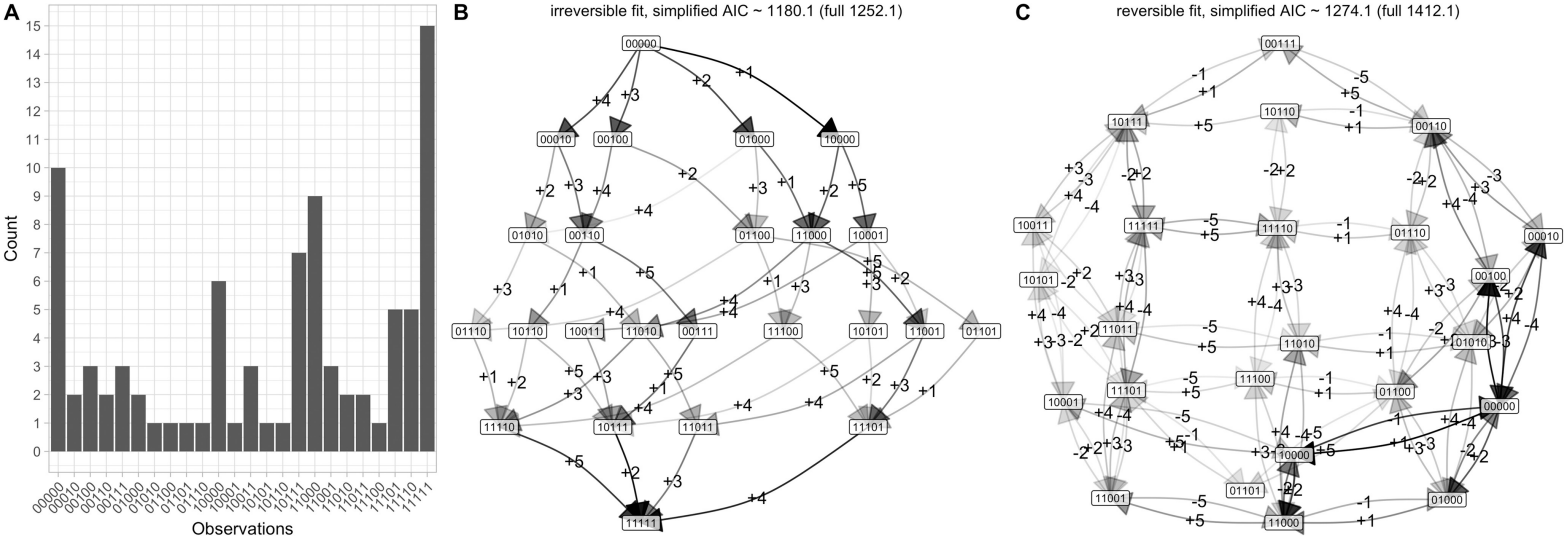
Accumulation dynamics in ovarian cancer progression. (A) The set of 87 cross-sectional observations of chromosomal aberrations in ovarian cancer (from independent patients, not linked by a phylogeny) from Knutsen *et al.* (2005). The (reduced) set of aberration locations is (8q+, 3q+, 5q−, 4q−, 8p−). (B–C) Inference of transition graphs using (B) irreversible and (C) reversible models.

Although reversibility of genetic changes in cancer progression is likely less important than in other systems, we also include a case study of cancer progression to connect with previous studies. We use an old but commonly-used dataset on chromosomal aberrations accumulating during ovarian cancer progression (Knutsen *et al.* 2005). These samples are taken from different patients and we therefore use the cross-sectional implementation of HyperMk. We focus on *L *=* *5 features, labelled by chromosome, chromosomal arm, and gain or loss of genetic material: 8q+ means a gain of material q arm of chromosome 8. In the order presented in the data, the feature set is (8q+, 3q+, 5q−, 4q−, 8p−) (Fig. 4A shows the frequency distribution of the chromosomal aberration combinations).

The most common pathways consistently identified by previous analyses have 8q+ and 3q+ as first steps, with 5q− and 8p− as competing early next steps and 4q− occurring on average somewhat later. Average orderings from HyperHMM (Moen and Johnston 2023) place 8q+ with the highest probability first (lower probability 5q− and 3q+), 3q+ second (lower probability 8p− and 5q−), 8p−, 4q−, or 8q+ third, 5q−, 4q−, or 8q+ fourth, and 8p− fifth (lower probability 5q−). These patterns are all borne out by both the reversible and irreversible inferred transition graphs (Fig. 4B and C), which support several pathways of progression dominated with 8q+ and 3q+ as first steps, with the other transitions occurring in consistent orderings thereafter.

Agreeing with the intuition that reversible transitions may play a limited role in determining cancer progression pathways (see Section 4), the AIC of the irreversible model is lower than that of the reversible model, suggesting that accumulation-only dynamics is the preferred picture for this dataset. The loss transitions supported by the inferred reversible model largely mirror acquisition transitions with lower probability (e.g. a lower probability −1 transition from 10000 to 00000 mirroring the +1 transition from 00000 to 10000), suggesting that unmatched loss transitions do not play a substantial role in determining dynamics (unlike, e.g. the loss of feature 1 from multiple different states in the synthetic reversible example above, Fig. 3).

### 3.3 Anti-microbial resistance

**Figure 5.**
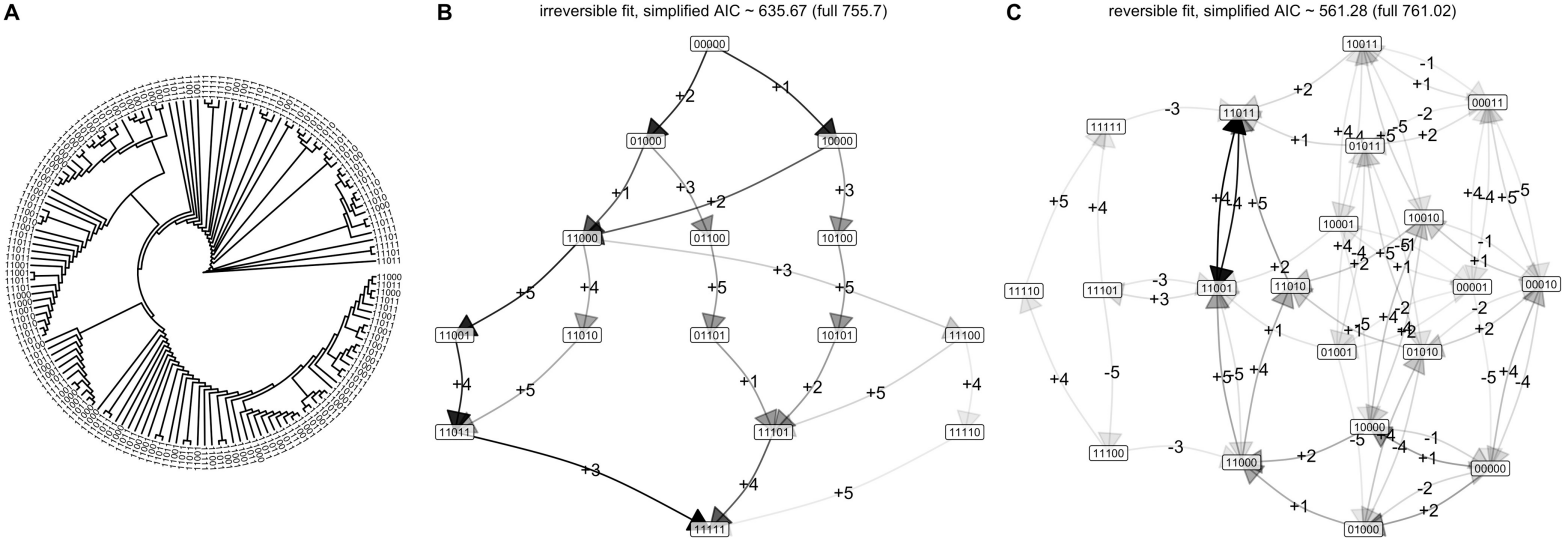
Evolutionary dynamics in tuberculosis drug resistance. (A) Observations of patterns of drug resistance from Casali *et al.* (2014). The phylogeny is plotted with uniform branch lengths for legibility; the true phylogeny has heterogeneous branch lengths. The (reduced) set of drug features is (INH, RIF, STR, EMB, PZA); 0 corresponds to susceptibility, 1 corresponds to resistance. (B–C) Inference of transition graphs using (B) irreversible and (C) reversible models.

To demonstrate the application of HyperMk to real-world questions, we consider data on the evolution of anti-microbial resistance in *Mycobacterium tuberculosis* (TB) (Casali *et al.* 2014), as curated in (Greenbury *et al.* 2020). Here, we take 150 isolates of TB with associated profiles of drug resistance, describing susceptibility (absence) or resistance (presence) to a collection of drugs. The observations are phylogenetically connected via a tree that was characterized using genetic data in the original publication (Fig. 5A). For this illustrative case, we consider the first *L *=* *5 of these drugs: isoniazid (INH), rifampicin/rifampin (RIF), streptomycin (STR), ethambutol (EMB), pyrazinamide (PZA). Previous work using accumulation-only models HyperTraPS and HyperHMM (Greenbury *et al.* 2020; Moen and Johnston 2023) have shown that of this set, INH and RIF are the most likely first acquisitions, followed by EMB then STR.

Following HyperMk fitting, this ordering is clearly visible in both reversible and irreversible models (Fig. 5B and C). The reversible model experiences a clear advantage according to AIC (561.3 versus 635.7 for the reversible and irreversible models, respectively), with the transition graph showing substantial probability flux associated with the loss of STR and EMB resistances.

### 3.4 Implementation

**Figure 6.**
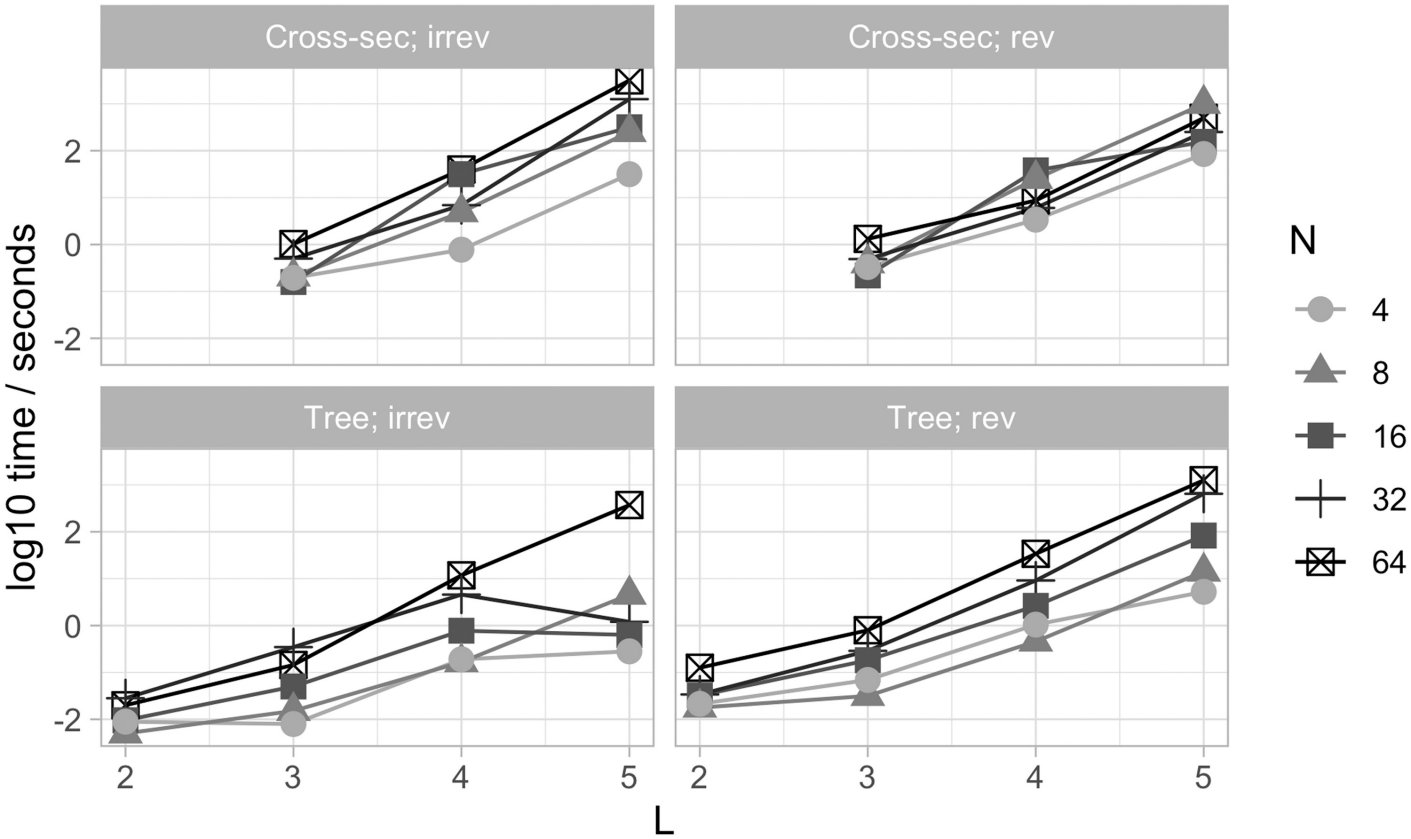
Timing for synthetic case studies. Simulated fits using *castor* to implement HyperMk. Data are randomly generated, either from a uniform samples of states (cross-sectional examples) or from a simulated tree and a reversible generating process as in Fig. 3A. Timings are for a single 3.2 GHz core (Apple M1 Pro).

The runtime of this approach, implemented using *castor*, does not scale particularly tractably with the number of features *L*. Figure 6 shows runtime in seconds for a fit involving *N* observations each of *L* features, on a simulated tree as in Fig. 3. Every additional feature multiplies the runtime by at least one order of magnitude, and sometimes approaching two. Feature sets larger than *L = *7 will take inconveniently long to infer with this implementation. However, as discussed below, this implementation uses both a “full” parameterization where no reduction of parameter space is used and a general Mk model fitting package. Indeed, one key contribution of this article is showing the connection between the different problems represented in Fig. 1, something that is evidenced by using off-the-shelf Mk software for all of them. A custom implementation for specific problems, involving, e.g. only pairwise interactions between features and/or specific speedups from the hypercubic transition network structure, may afford substantial gains in speed. As described in Section 2, several different existing packages in addition to *castor* provide functionality for fitting Mk models compatible with this accumulation modelling picture (Supplementary Fig. S2).

We observed that numerical convergence is a consistent issue with these approaches. Intuitively, the presence of reversible transitions means that an infinite set of possible pathways can in principle be responsible for a given observation. While in practice any non-infinite transition rates will act to decrease the probability of longer pathways, this dramatic expansion of possibilities over the irreversible case makes inference with the reversible model numerically challenging. We found that a combination of multiple start points and looser constraints on the number of permitted iterations mitigated these challenges to some extent—at the inevitable cost of more computational resource. There were also some library-specific issues related to this convergence: the Mk model fit with *castor* sometimes crashed when multiple start points were invoked. Our working approach—implemented in the software accompanying this article—is thus to avoid multiple start points and only parallelize over different experiments; the case studies here in Figs. 3–5 are all tractable on a single machine, with the tuberculosis case study taking the longest (several hours) to converge.

## 4 Discussion

We have introduced and demonstrated a way of supporting reversible transitions in accumulation modelling using the Mk model. Of course, the value of supporting reversibility depends on the scientific question being asked. In cancer progression, mutations occur and clonally proliferate as cells divide. Depending on what the “entity” being modelled is—a cell, a tumor, a patient, etc. (Diaz-Uriarte and Johnston 2024)—reversibility may simply be inappropriate to consider. The probability of a reverse mutation “undoing” a particular mutation at the cellular level is presumably very low, although at the tumor level it is not inconceivable that cell-level selection could remove a mutation from the cellular population once it has arisen.

In evolution, e.g. the evolution of anti-microbial resistance, this question can be case-specific. AMR traits in *Klebsiella pneumoniae* are often acquired *via* transfer of plasmids—mobile DNA elements—which can be gained and very readily lost again (Holt *et al.* 2015). AMR traits in tuberculosis are often acquired *via* chromosomal mutations, which are less readily lost once they have been acquired (Casali *et al.* 2014). At the level of single cells, reversibility may therefore be more important to consider for Klebsiella than tuberculosis (Renz *et al.* 2024). And—in agreement with Fig. 5—reversible dynamics may occur at the population level (one strain outcompeting another to be the dominant observed type) without requiring single-cell reversibility (Diaz-Uriarte and Johnston 2024). In other cases, reversibility may be simply ill-posed—e.g. in accumulation modelling of students completing tasks in online courses (Peach *et al.* 2021).

We have not focused on a continuous time variable in this study, instead viewing dynamics as “ordinal” and reporting the sequence, rather than timing, of transitions. This approach mirrors HyperTraPS and HyperHMM (Johnston and Williams 2016, Greenbury *et al.* 2020, Moen and Johnston 2023), but many other approaches (including a recent generalization of HyperTraPS (Aga *et al.* 2024)) assume a continuous timescale that is implicitly or explicitly connected to the observations (Schill *et al.* 2020, Diaz-Uriarte and Herrera-Nieto 2022, Diaz-Uriarte and Johnston 2024, Schill *et al.* 2024). The Mk model approach described here has the full capacity to capture continuous timing in observations, as the branch lengths in the phylogeny (or in the set of synthetic trees, for cross-sectional data) correspond to times between states. The rate parameters learned in the model fitting then become true rates in the time behavior of the system, rather than just describing relative propensities of events. Accumulation problems where continuous time data are available can exploit this natural feature of the Mk approach.

A clear shortcoming in this implementation is the number of features that can be considered. Existing methods for accumulation modelling can deal with dozens, and sometimes over a hundred, interacting features—although the case of arbitrary dependencies between sets of features, not just pairwise interactions, is more limited (see details and discussion in Aga *et al.* (2024)). Here, feature sets larger than *L *=* *7 approach computational intractability. However, it is very possible that different software implementations will change this limitation—and future developments exploiting the restrictions on parameter space specific to the reversible accumulation model may substantially increase efficiency. Approaches for reducing dimensionality in accumulation modelling datasets are also under development (Dauda *et al.* 2024). In particular, the picture outlined in this research allows arbitrary independent transitions between states, rather than coarse-graining transition parameters by the features involved in the transition (Fig. 1). More restricted parameterizations, e.g. reflecting the pairwise-only interactions used in the *L*^2^ parameterization of HyperTraPS and equivalent in mutual hazard networks, would lead to smaller parameter spaces and presumably faster inference. The *corHMM* package (Boyko and Beaulieu 2021) allows different classes of parameter structure to be specified; implementations of accumulation modelling for single trees (or a more general picture, with future development) may provide a straightforward solution for this adaptation.

More generally, connections between the evolutionary and systematic literatures and accumulation modelling may allow more transfer of promising insights between the fields. One scenario that is likely to become more prevalent with single-cell sequencing involves data sets where several patients provide multiple within-patient samples which are phylogenetically related (Caravagna *et al.* 2018, Luo *et al.* 2023, Aga *et al.* 2024) which naturally fit in the hypercubic Mk modelling framework. A broader common question in applied accumulation modelling is whether continuous and/or ordinal features, as well as the presence/absence features, can be analyzed in the same framework. While binary accumulation approaches have been used to analyze datasets containing ordinal features (e.g. the levels of coma presentation in malaria patients (Johnston *et al.* 2019)), this has typically been done with a rather crude coding approach involving inequalities (a set of *n* features denoting “presence” of *x *<* *1, *x *<* *2, *x *<* *3, and so on). A recent advance in the systematics literature provides an approach by which the coupled dynamics of discrete and continuous characters can be naturally analyzed (Boyko *et al.* 2023). Broadly, we anticipate that if the usual picture of data linked by a dichotomous phylogenetic tree with branch lengths can be relaxed—to star phylogenies or sets of trees or “stumps” for example—a wide range of approaches from phylogenetic methods will find use in the analysis of accumulation processes.

## Supplementary data

Supplementary data are available at *Bioinformatics* online.

##  

Conflict of interest: No direct economic conflicts of interest declared.

## Funding

This project has received funding from the European Research Council (ERC) under the European Union’s Horizon 2020 Research and Innovation Programme [Grant agreement No. 805046 (EvoConBiO) to I.G.J.]. I.G.J. was supported by the Trond Mohn Foundation [project HyperEvol under grant agreement No. TMS2021TMT09], through the Centre for Antimicrobial Resistance in Western Norway (CAMRIA) [TMS2020TMT11]. R.D.U. was supported by grant PID2019-111256RB-I00 funded by MCIN/AEI/10.13039/501100011033.

## Supplementary Material

btae737_Supplementary_Data

## Footnotes

1 The *castor* package also supports bootstrap resampling and multiple, parallelised fitting attempts per bootstrap, though we have not made use of it here.
