# Supplementary material for: A hypercubic Mk model framework for capturing reversibility in disease, cancer, and evolutionary accumulation modelling
Source: Bioinformatics. 2024 Dec 12;41(1):btae737. doi: 10.1093/bioinformatics/btae737 (PMC11681934; doi:10.1093/bioinformatics/btae737)
Supplement: btae737_Supplementary_Data [file btae737_supplementary_data.pdf]

A hypercubic Mk model framework for capturing reversibility in disease, cancer, and evolutionary accumulation modelling

Iain G. Johnston and Ramon Diaz-Uriarte

Supplementary Information

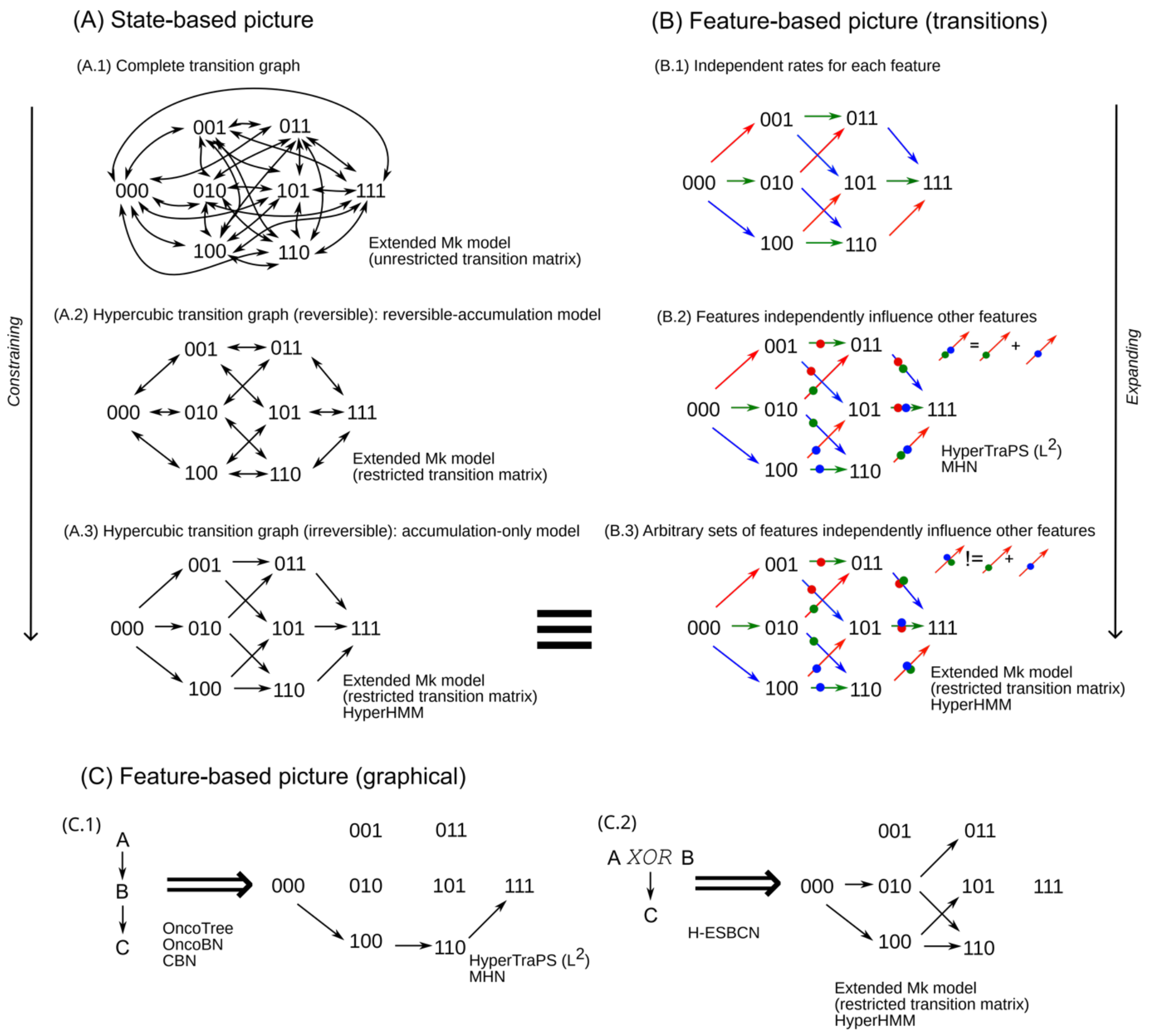

Supplementary Figure 1. **State spaces and features in (reversible) accumulation and Mk models.** (A) Transitions supported in increasingly constrained modifications of the Mk model, with rates based on *states:* A.1 unrestricted dynamics, A.2 reversible accumulation, A.3 irreversible accumulation. (B) Increasingly flexible modifications of the accumulation model with rates based on *features and their interactions*. Model B.1 (L^1 rates) has the same rate (colour) for every transition involving a given feature; model B.2 (L^2 / mutual hazards) allows pairwise influences between features, so that having acquired feature X can influence the rate of acquiring feature Y (denoted by a coloured ball for each acquired feature); model B.3 allows arbitrary contributions to all feature rates from any subset of acquired features. Models A.3 and B.3 are equivalent. (C) Correspondence between the Directed Acyclic Graph (DAG) representation of feature-based models and their hypercubic representation. In C.1, the DAG describing the dependence of feature acquisitions is linear: C depends on B, which depends on A. In C.2, C depends A and B via a logical XOR relationship. This panel also shows two common alternative notations: letters to denote the altered (mutated) feature in DAGs vs. 0/1 to indicate non-altered/altered for the corresponding feature in hypercubic graphs.

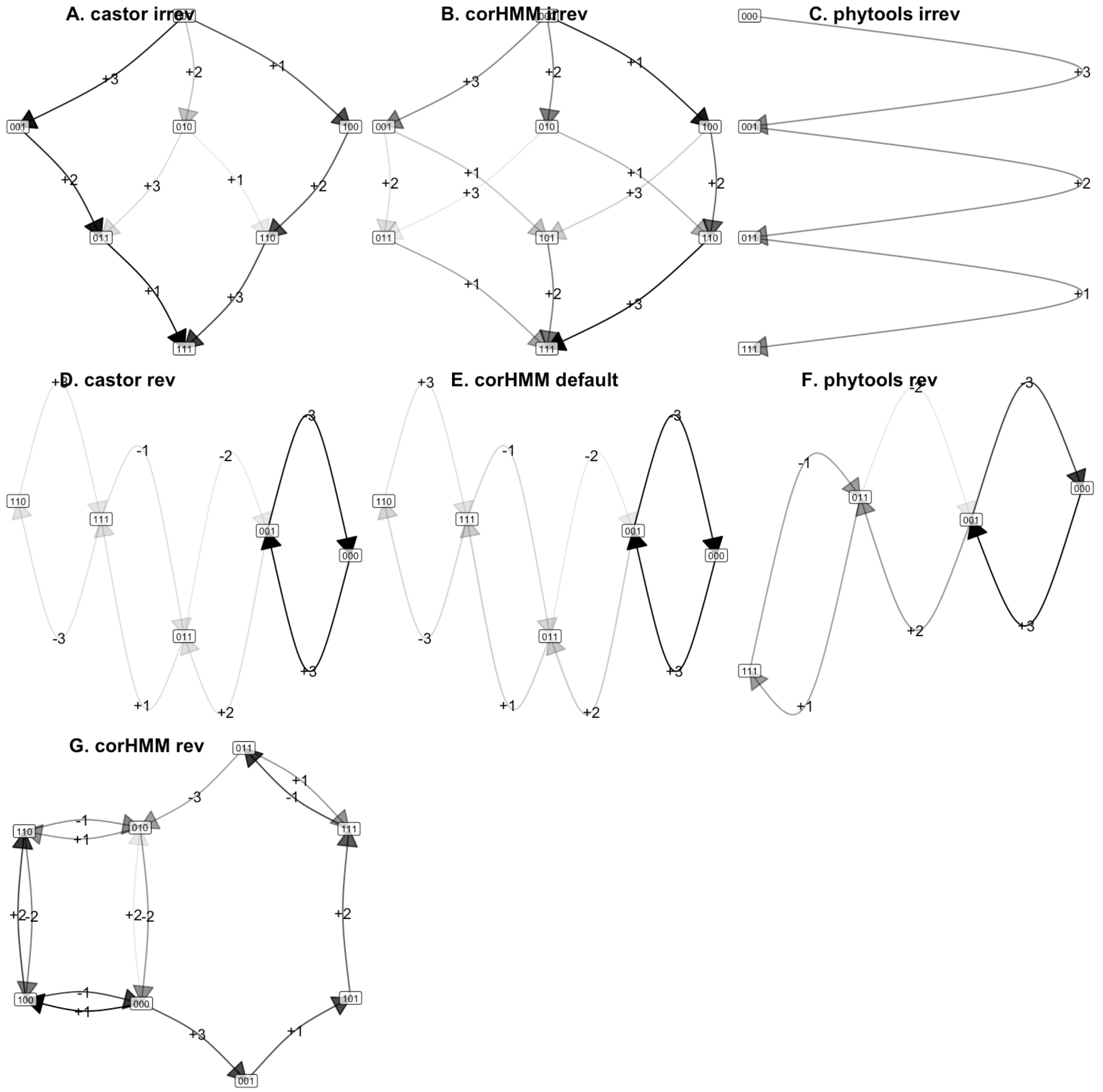

Supplementary Figure 2. **Different Mk model fitting approaches.** Inferred transition networks for synthetic data capturing a single pathway with reversible dynamics (observations 000, 001, 011, 111, 110). (A-C) irreversible model fits using the single pathway case study using *castor*, *corHMM*, and *phytools*; (D-F) reversible model fits. The *phytools* fit in this implementation seems to ignore the 110 observation. In (E) the default *corHMM* fit has been performed, which considers only states present in the observation set; in (B, G) the presence of all 2^L possible binary states has been enforced.

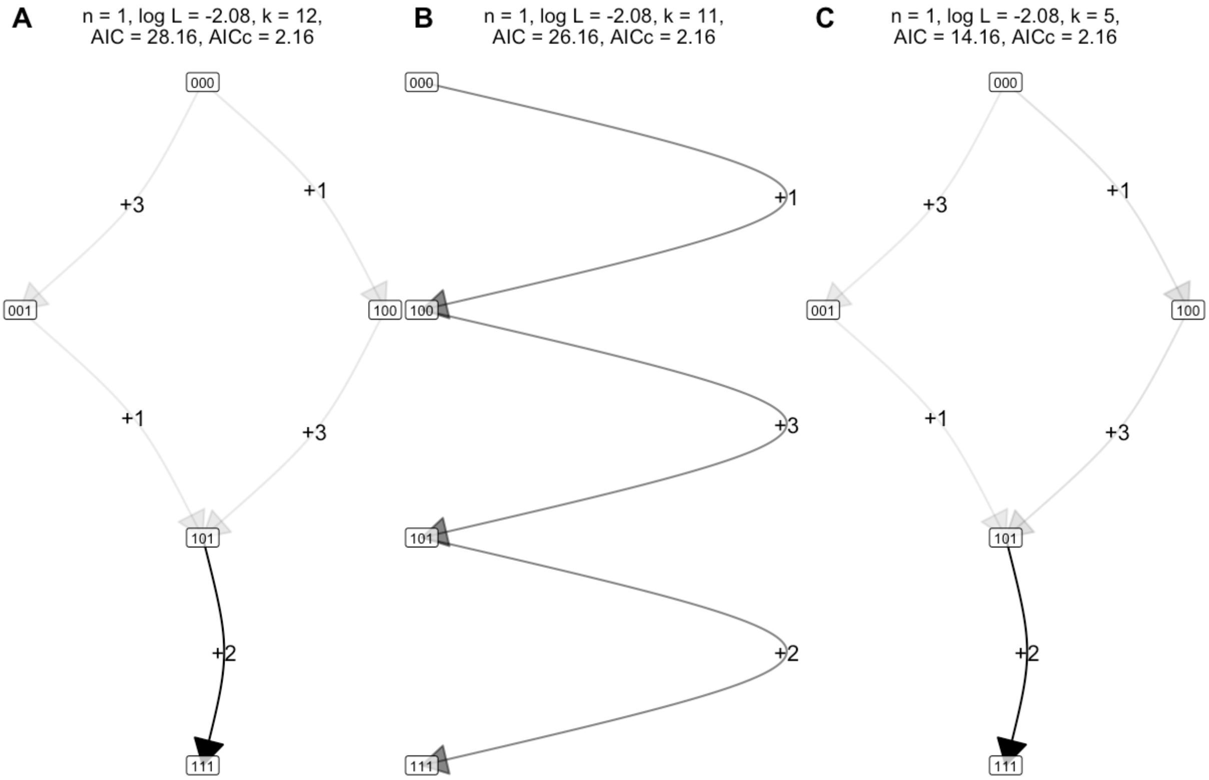

Supplementary Figure 3. **Illustration of "pruning" parameters.** (A) An unpruned fit of an irreversible L = 3 model to a single (n = 1) observation 101 involves a model with $k = L\,2^{L-1} = 12$ edge parameters. Only those 5 supporting nonzero flux in the fitted model are plotted here; the others are not identifiable from the data. Equal weight is given to the possibilities 000-001 and 000-100, both of which are compatible with the observation. (B) The edge parameter 000-001 has been removed and the model refitted; now the dynamics are completely constrained and the AIC is lowered by 2 (2× number of parameters removed). (C) All parameters corresponding to fluxes $< 10^{-4}$ in (A) have been removed and the model refitted. Differences to the inferred dynamics are negligible and the AIC is lowered by 14 (2× number of parameters removed) compared to (A). This method (C) is used throughout the study.

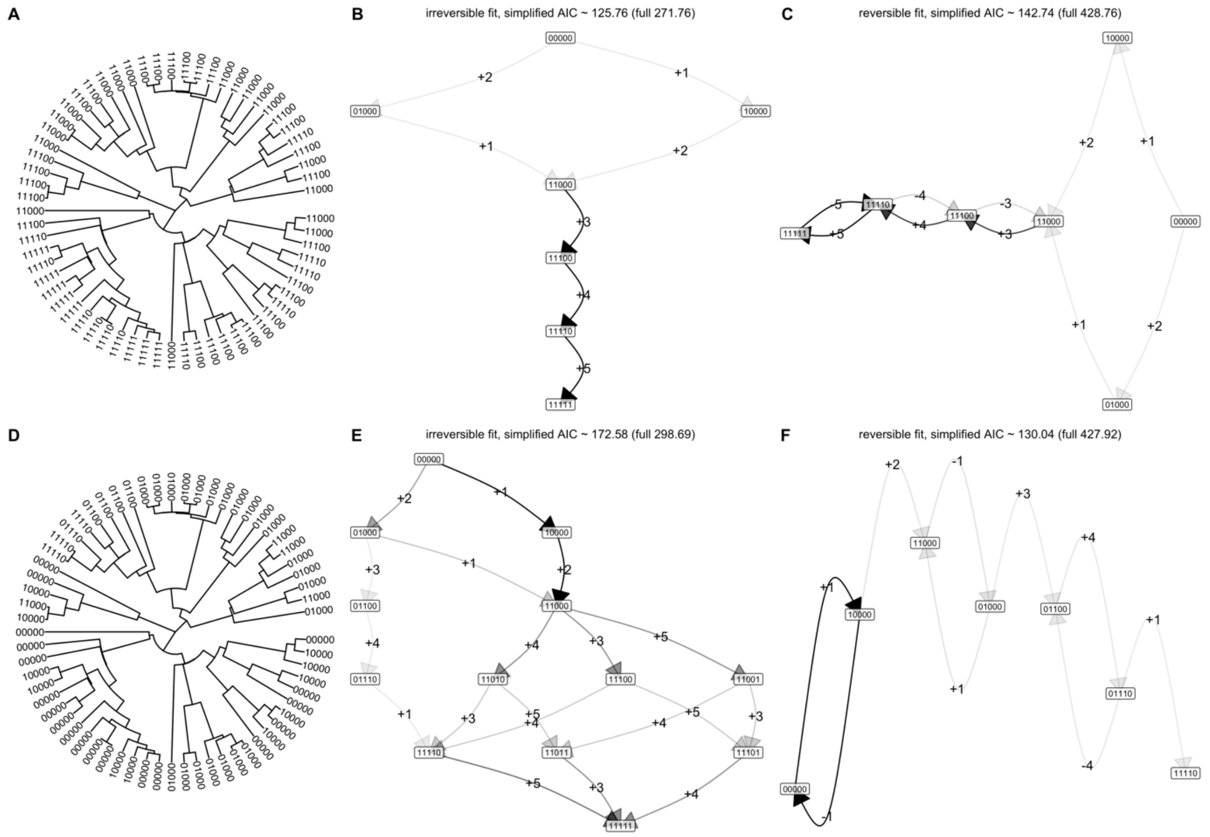

Supplementary Figure 4. **Capturing irreversible and reversible accumulation dynamics with the Mk model.** (A) Observations of different states of an irreversible process on the tips of a 64-node randomly-simulated tree. (B) Inferred transition graph for these data, using an irreversible model. Node labels give different states; edge labels give the feature that is gained (+) or lost (-) at each transition, but in this irreversible case no feature losses (-) are allowed. The darkness of an edge gives its rate: darker edges have higher associated transition rates. (C) Inferred transition graph for these data using a reversible model. The AIC values for the full fitted model, and for the simplified model discarding all edges that do not support flux from the 00000 state (i.e., edges with two or more transitions), are given: the AIC for the irreversible model is lower, reflecting the irreversible generating process. (D) Observations of different states of a reversible process on the tips of a 64-node randomly-simulated tree. Here, the first feature can be lost as well as gained. (E) irreversible and (F) reversible model transition graphs as in (B-C). In the reversible case, the "-1" edges corresponding to the loss of the first feature appear ubiquitously; the AICc value for the reversible model is now lower, reflecting the model's better ability to capture reversible dynamics.

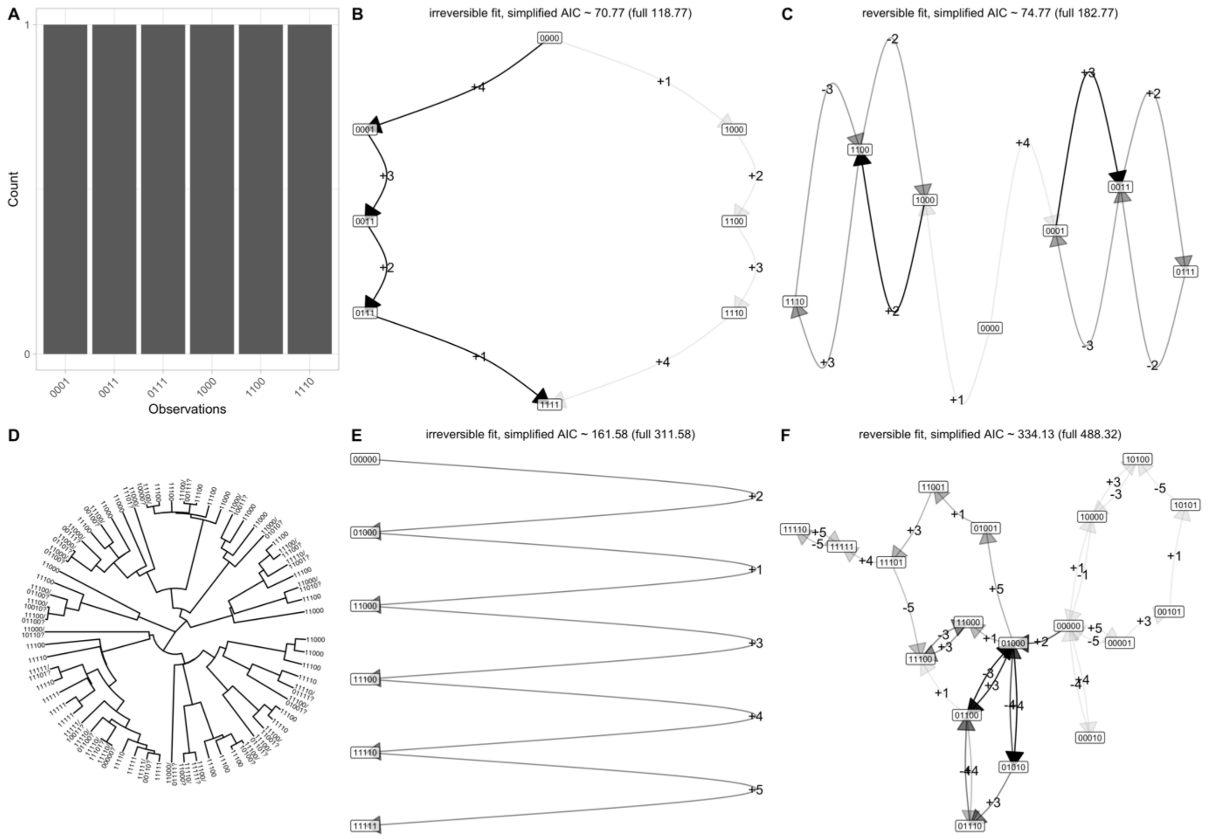

Supplementary Figure 5. **Capturing positive and negative influences, cross-sectional observations, and uncertainty with the Mk model.** (A) Cross-sectional observations generated by the two-pathway system described in the text. Now no tree links observations, which are taken to reflect independent samples. (B-C) Inference of transitions graphs for this cross-sectional case using (B) irreversible and (C) reversible models. In both cases, the competing pathways (features 4, 3, 2, 1 versus features 1, 2, 3, 4) are readily distinguished. (D) Observations on a simulated tree, where some observations are uncertain. Here, the single pathway (features 1, 2, 3, 4, 5) generates observations, but half of the observations (marked “?”) are confounded by mixing with another random state. (E-F) Inference of transition graphs for this uncertain case using (E) irreversible and (F) reversible models. The core pathway is recovered in both models but with some probability flux to other states supported.
